# Clinical Severity of *Clostridium difficile* PCR Ribotype 027: A Case-Case Study

**DOI:** 10.1371/journal.pone.0001812

**Published:** 2008-03-19

**Authors:** Oliver W. Morgan, Boaventura Rodrigues, Tony Elston, Neville Q. Verlander, Derek F. J. Brown, Jonathan Brazier, Mark Reacher

**Affiliations:** 1 East of England Regional Epidemiology Unit, Health Protection Agency, Cambridge, United Kingdom; 2 Eastern Deanery Public Health Training Scheme, Cambridge, United Kingdom; 3 Essex Rivers NHS Trust, Colchester, United Kingdom; 4 Statistics, Modelling and Bioinformatics Department, Centre for Infections, Health Protection Agency, London, United Kingdom; 5 Clinical Microbiology and Public Health Laboratory, Health Protection Agency, Addenbrookes Hospital, Cambridge, United Kingdom; 6 Anaerobe Reference Laboratory, National Public Health Service, University Hospital of Wales, Cardiff, United Kingdom; University Paris 7, France

## Abstract

**Background:**

*Clostridium difficile* is a leading infectious cause of health care associated diarrhoea. Several industrialised countries have reported increased *C. difficile* infections and outbreaks, which have been attributed to the emergent PCR ribotype 027 strain.

**Methods and Findings:**

We conducted a case-case study to compare severity of *C. difficile* disease for patients with 027 versus non-027 ribotypes. We retrospectively collected clinical information about 123/136 patients with *C. difficile* infections admitted to hospitals in the East of England region in 2006 and from whom stool isolates were cultured and ribotyped as part of an earlier national survey. We defined severe *C. difficile* disease as having one or more of shock, paralytic ileus, pseudo membranous colitis or toxic megacolon. Patient median age was 83 years old (range 3 to 98, interquartile range 75 to 89), 86% were prescribed antibiotics in the eight weeks before illness onset, 41% had ribotype 027 and 30-day all cause mortality during hospital admission was 21%. Severe disease occurred in 24% (95%CI 13% to 37%) and 17% (95%CI 9% to 27%) of patients with PCR ribotype 027 and non-027 ribotypes respectively. In a multivariable model, ribotype 027 was not associated with severe disease after adjusting for sex, discharge from hospital prior to 60 days of current admission, gastroenteritis on admission, number of initiator antibiotics for *C. difficile* disease, and hospital where the patient was admitted.

**Conclusions:**

Our study found no evidence to support previous assertions that ribotype 027 is more virulent than other PCR ribotypes. This finding raises questions about the contribution of this strain to the recent increase in *C. difficile* disease throughout North America and Europe.

## Introduction

*Clostridium difficile* is a Gram-positive spore forming anaerobic bacterium that is found in the normal gut flora of man. *Clostridium difficile* associated disease (CDAD) generally follows ingestion of antibiotics that leads to selection of toxin-producing strains, resulting in a leading infectious cause of health care associated diarrhoea [1]. CDAD ranges from mild uncomplicated diarrhoea to severe diarrhoea complicated by one or more of fluid loss, shock, leukocytosis, paralytic ileus, pseudomembranous colitis, and toxic megacolon, and sometimes death [2]. Prevention and control of CDAD crucially depends on maintaining high levels of institutional hygiene, including the prompt recognition and isolation of individuals with application of enteric precautions, and on minimising exposure to antibiotics [1].

Molecular typing of toxigenic strains of *C. difficile* based on detection of genes encoding toxins A and B within the pathogenicity locus (*PaLoc*) [3], [4] has led to the recognition of at least 22 distinct toxinotypes [3], [5]. Health systems in a number of industrialised countries have reported recent increases of *C. difficile* infections and outbreaks have been attributed to the emergence of a strain characterised as toxinotype III, North American pulsed-field type 1, PCR ribotype 027 [6], [7], [8], [9], [10]. It has been asserted that this strain is more virulent than other strains [3], [11], a notion supported by very high levels of toxin A and B production in vitro [11]. It is possible, however, that the impression of greater virulence of the 027 ribotype could reflect, at least in part, biases in the sampling, testing and reporting of cases. In this study, we examine whether patients with CDAD due to ribotype 027 had more severe disease than patients with CDAD caused by other ribotypes.

## Methods

### Study Design

We conducted a case-case study. This study design is a variant of the case-control design whereby only cases with the disease (in this case *C. difficile*) are selected for the study [12]. Cases are grouped by subtype of infectious disease organism, in this case *C. difficile* 027 versus non-027 ribotypes, and their outcomes (here we consider clinical severity of disease) are compared. The advantage of using a case-case design is that it frequency matches on all aetiological factors, both known and unknown, and selects groups that are similar for disease-specific risk factors [13]. In this study, a case-case design provides a non-biased comparison of disease severity among patients with different strains of *C. difficile*.

### Identification of Patients

We retrospectively identified inpatient cases of *C. difficile* from 16 National Health Service (NHS) hospitals in the East of England region included in a national survey of *C. difficile* PCR ribotypes, as reported elsewhere [14], [15]. The survey selected all patients with CDAD detected by microbiology laboratories in the East of England during one allocated week between 9 January and 3 March 2006. Stool isolates from these patients were sent to the regional coordinating laboratory where anaerobic culture was undertaken. PCR ribotyping was done by the Health Protection Agency Anaerobe Reference Laboratory in Cardiff.

### Data Collection

We developed a structured proforma to extract information from medical records about patients' demographic details; main diagnosis at admission; treatment during 8 weeks prior to diagnosis of suspected CDAD with antibiotics, H2 agonists and proton-pump inhibitors; *C. difficile*-related illness; and all cause mortality during hospital admission within 30-days of onset of CDAD. Data were extracted by medical microbiologists involved in patient care or by a member of the study team. Individuals who extracted data were not aware of the ribotyping results. Data were double-entered, compared and corrected using EpiData (v.3.1) software [16].

### Analysis

We defined severe CDAD as having one or more of shock (systolic BP 100 mmHg or less at any time, and/or oliguria), paralytic ileus, pseudo membranous colitis or toxic megacolon. We considered the following risk factors: infection with 027 or non-027 ribotypes, age group (by quintile), sex, previous discharge from any hospital within 60 days prior to admission, having gastroenteritis at admission, being immunocompromised, use of proton pump inhibitors or H2 agonists within 8-weeks before diagnosis of CDAD, use of antibiotics in the 8-weeks before diagnosis of CDAD (where glycopeptides and metronidazole were considered protective against CDAD and all other antibiotics were considered as potential initiators of CDAD), and the hospital to which the patient was admitted.

We conducted a single variable analysis wherein each risk factor was examined for its association with severe CDAD. Variables with probability p<0.3 in the single variable analysis were then entered into a multivariable logistic regression model. The variable for hospital was included as a random effect in the analysis; all other variables were analysed as fixed effects. Analysis was done using STATA 9.1 [17].

### Ethical Approval

The study protocol was approved by the Cambridge Local Research Ethics Committee (Ref: 06/Q0108/249).

## Results

**Figure 1:**
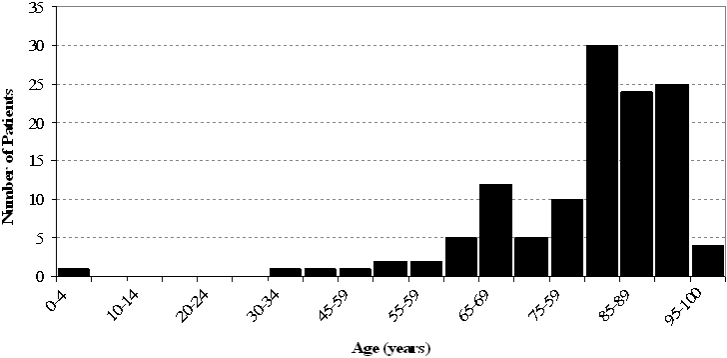
Age of patients with *Clostridium difficile* associated disease, East of England, 2006

There were 136 patients admitted to a hospital in the East of England with CDAD and from whom an isolate was included as part of the national survey of *C. difficile*. We were able to obtain clinical information for 123 patients (90%). There were slightly fewer males than females (Table 1). The median age was 83 years old, with a range of 3 to 98 years old and interquartile range of 75 to 89 years old (Table 1). The age distribution was skewed towards the older ages (Figure 1). The most frequent diagnoses at admission were gastrointestinal, respiratory, central nervous system, urinary and renal complaints, cardiovascular and trauma (Table 1). Twelve percent of patients (n = 13/112) were immunocompromised and 55% (n = 54/98) had previously been discharged from hospital within 60 days of the current admission (Table 1). PCR ribotype 027 was identified in 41% (n = 51/123) of patients (Table 1). Severe disease was experienced by 20% (n = 24/123) of patients (Table 1). A fifth (n = 25/117) of patients died (all causes) within 30-days of hospital admission (Table 1).

**Table 1:** Characteristics of patients with *Clostridium difficile* associated disease, East of England, 2006

Patient Characteristics	Number	(%)
**Demographic features [N = 123]**		
Males	55	(44)
Median age in years (interquartile range)	83	(75 to 89)
**Main diagnosis at admission [N = 123] ** *		
Gastrointestinal **	26	(21)
Respiratory	20	(16)
Central Nervous System	19	(15)
Urinary & Renal	15	(12)
Cardiovascular	13	(11)
Trauma	12	(10)
Malignancy	9	(7)
Metabolic	4	(3)
Psychiatric	3	(2)
Skin	3	(2)
Muscular skeletal	3	(2)
Diabetes	3	(2)
Genital	0	-
Unspecified	16	(13)
Missing	5	(4)
**Immunocompromised [N = 112] ** †		
Yes	13	(12)
No	99	(88)
**Discharged from hospital prior to 60 days of current admission [N = 98] ** §		
Yes	44	(45)
No	54	(55)
**PCR Ribotype 027 [N = 123]**		
Yes	51	(41)
No	72	(59)
**Severe Disease [N = 123] ** ‡		
Yes	24	(20)
No	99	(80)
**30-day all cause mortality during hospital admission [117] ** §§		
Yes	25	(21)
No	92	(79)

* Some patients had more than one diagnosis at admission

** 3 patients were admitted with CDAD

† Data missing for 11 patients

‡ Severe CDAD defined as one or more of shock, paralytic ileus, pseudo membranous colitis or toxic megacolon

§ Data missing for 25 patients

§§ Data missing for 6 patients

Information about frequency of diarrhoea was recorded in the medical records for 58 patients, of whom 78% (n = 45) had 3–5 stools per day and 22% (n = 13) has six or more stools per day. Thirty four percent of patients (n = 30/87) had abdominal pain, while only 10% (n = 8/78) had blood in the stool. Fever was present in 13% (n = 13/102) of patients. Leukocytosis was recorded for 48% (n = 52/108) of patients. Nineteen percent of patients had shock (n = 21/112), 3.7% (n = 4/107) had paralytic ileus, 4% (n = 4/100) pseudomembranous colitis and one patient had toxic megacolon.

In the eight weeks before onset of CDAD, 86% of patients had been prescribed an antibiotic, with cephalosporins and quinolones most frequently used, followed by penicillins, metronidazole and macrolides (Table 2). Only 2% had been prescribed an antibiotic protective against CDAD, while 51% had received other classes of antibiotic (Table 2). Both initiating and protective antibiotics were prescribed to 33% of patients. The number of classes of initiating antibiotics taken in the eight weeks prior to onset of CDAD was zero in 16% of patients, one in 20%, two in 31%, three in 15% and four or more in 18% (Table 2). Proton pump inhibitors were prescribed to about a third of patients while only 9% received an H2 antagonist (Table 2).

**Table 2:** Treatment history for patients with *Clostridium difficile* associated disease, East of England, 2006

Treatment History	N	%
**Antibiotics used 8 weeks before CDAD** * ** [N = 123]**		
Any antibiotic	106	(86)
Cephalosporins	57	(46)
Quinolones	53	(43)
Penicillins	47	(38)
Metronidazole	38	(31)
Macrolides	23	(19)
Trimethroprim	17	(14)
Glycopeptides	10	(8)
Carbapenems	6	(5)
Aminoglycosides	2	(2)
Nitrofurantoin	1	(1)
**Protective and Initiating antibiotics used in 8 weeks before CDAD** ** **[N = 123]**		
Protective	3	(2)
Initiating	63	(51)
Both	40	(33)
None	17	(14)
**Number of Initiating antibiotics used in 8 weeks before CDAD ** ** ** [N = 123]**		
0	20	(16)
1	24	(20)
2	38	(31)
3	19	(15)
4	10	(8)
5	11	(9)
6	1	(1)
**Proton pump inhibitors [N = 109] ** †		
Yes	42	(39)
No	67	(61)
**H2 antagonists [N = 89] ** ‡		
Yes	7	(9)
No	82	(92)

* Some patients were prescribed antibiotics from more than one class

** Protective antibiotics: glycopeptides and metronidazole

† Data missing for 14 patients

‡ Percentages do not sum to 100% due to rounding. Data missing for 34 patients

Table 3 shows the proportion of patients with non-severe and severe CDAD by C.*difficile* PCR ribotype. Of patients with ribotype 027, 24% had severe CDAD compared to 17% of patients with non-027 ribotypes. The 95% confidence intervals (CI) for these two groups overlapped and included both point estimates, indicating that they were not statistically different. Results from the single variable analysis are shown in Table 4 and ribotype 027, sex, discharge from hospital within 60 days of current admission, gastroenteritis on admission, number of initiator antibiotics for CDAD, and hospital where the patient was admitted were included in the model. The result of the multivariable model for CDAD severity is shown in Table 5. Only sex showed a statistically significant association, with females less likely to have severe disease compared to males.

**Table 3:** Proportion of patients with severe *Clostridium difficile* associated disease and deaths during admission (all causes) by PCR ribotype 027

Severity of *Clostridium difficile* associated disease					
	Number of patients			% Severe	95% CI
PCR ribotype 027	Non-severe	Severe	Total		
Yes	39	12	51	24	13 to 37
No	60	12	72	17	9 to 27
Total	99	24	123	20	13 to 28

**Table 4:** Single variable analysis of risk factors for severe *Clostridium difficile* associated disease (CDAD), East of England, 2006

		Clinical Severity				
		Severe	Not Severe	Odds Ratio	95%CI	P-value
**Sex**	Male	14	41			0.14*
[N = 123]	Female	10	58	0.50	0.20 to 1.25	
**Age group (years)**	3–68	6	18			0.9
[N = 123]	69–79	5	18	0.83	0.22 to 3.23	
	80–84	4	19	0.63	0.15 to 2.61	
	85–89	4	20	0.60	0.15 to 2.47	
	90–98	5	24	0.63	0.16 to 2.37	
**Discharged from hospital prior to 60 days of current admission**	No	8	46			0.21*
[N = 98]	Yes	11	33	1.92	0.69 to 5.29	
**Immunocompromised**	No	16	83			0.9
[N = 112]	Yes	11	2	0.94	0.19 to 4.67	
**PCR ribotype 027**	No	12	60			0.3
[N = 123]	Yes	12	39	1.54	0.63 to 3.77	
**Proton pump inhibitors or H2 antagonists used in 8-weeks before CDAD**	No	12	51			0.9
[N = 109]	Yes	9	37	1.04	0.39 to 2.71	
**Gastroenteritis on admission**	No	16	81			0.12*
[N = 123]	Yes	8	18	2.25	0.84 to 6.06	
**Protective and inciting antibiotics used in 8-weeks before CDAD**	Protective	1	2			0.8
[N = 106]	Inducing	15	48	0.63	0.05 to 7.39	
	Both	8	32	0.5	0.04 to 6.23	
**Number of initiator antibiotics used in 8-weeks before CDAD**	0	1	19			0.22*
[N = 123]	1	6	18	6.33	0.69 to 57.9	
	2	9	29	5.9	0.69 to 50.4	
	3+	8	33	4.61	0.53 to 39.7	
**Hospital Trust** [N = 123]	Proportion of total variance contributed by Trust variance			0.16	0.03 to 0.53	0.034*

* Included in the multivariable model

**Table 5:** Multivariable logistic regression model of risk factors for severe *Clostridium difficile* associated disease (CDAD), East of England, 2006

Risk Factor		Odds Ratio	95% CI	p-value
PCR ribotype 027	No	1.00		
	Yes	2.07	0.63 to 6.81	0.23
Sex	Male	1.00		
	Female	0.26	0.08 to 0.89	0.03
Discharged from hospital prior to 60 days of current admission	No	1.00		
	Yes	1.92	0.56 to 6.61	0.3
Gastroenteritis admission	No	1.00		
	Yes	1.40	0.31 to 6.3	0.7
Number of initiator antibiotics before CDAD	0			0.8
	1	2.64	0.2 to 34.3	
	2	3.31	0.29 to 38.1	
	3+	2.14	0.17 to 26.5	
Hospital	Proportion of total variance contributed by trust variance	0.13	0.01 to 0.73	0.18

## Discussion

We did not find evidence to suggest that patients infected with *C. difficile* PCR ribotype 027 were more likely to have severe disease than patients infected with other PCR ribotypes. In a multivariable model, men were more likely to have severe disease than women. The number of antibiotics prescribed in the 8-weeks prior to diagnosis of CDAD was not associated with greater disease severity.

### Strengths and Limitations

Selection bias in our patient sample was minimised as recruitment was done without reference to ribotype or disease severity. Patients for whom we could not obtain medical records (10%) were more likely to have died. However, the proportion with ribotype 027 was similar to patients included in our study and is unlikely to have seriously biased our results. We reduced bias in the measurement of CDAD severity by ensuring that individuals who extracted clinical data had no prior knowledge of PCR ribotype.

No standard definition for severe CDAD exists, although several have been proposed [1], [18]. To minimise misclassification bias we used a conservative definition for categorising patients as severe and less severe. Retrospective extraction of data from medical records led to some missing data in this study, especially for frequency of diarrhoea. Nevertheless, reviewing medical records is likely to provide a more accurate picture of existing medical practice than data collected during prospective studies.

### Interpretation of Results

Our study provides a snapshot of patients with CDAD in hospitals in the East of England region in 2006. While this patient group predominantly consisted of the elderly, a notable proportion (11%) of patients were under 65 years old, highlighting that CDAD can occur in all age groups. High antibiotic ingestion (84% of our patients had received at least one antibiotic in the 8-weeks prior to onset of CDAD) is a cause for concern, given that development of CDAD is recognised to generally follow exposure to antibiotics. This reiterates the need for concerted efforts to limit exposure to unnecessary antibiotics. We found that a higher proportion of men had severe CDAD, possibly because they had more severe underlying illness on admission to hospital, although we were unable to consider this in our analysis. We also observed that 41% of our patient population was infected with PCR ribotype 027 compared to about 25% of CDAD patients in England as a whole [14]. This may be due to geographical clustering resulting from colonisation of hospitals with specific strains [19]. Thirty day all cause mortality during hospital admission was 21% in our study, which is similar to other studies that report mortality rates ranging from 11% to 25% [19], [20], [21], [22].

Few studies have considered whether specific strains of *C. difficile* cause more severe disease. Loo *et al* conducted a prospective study of an outbreak of *C. difficile* in 12 hospitals in Quebec, Canada [22]. Severe CDAD (defined as a patient who died within 30 days of CDAD diagnosis and where *C. difficile* contributed to death, if the patient had a colectomy or required admission to the intensive care unit because of CDAD) occurred in 16.7% (n = 22/132) of patients with isolates that had both binary toxin and a partial deletion in the *tcdC* gene (which represses production of toxin A and toxin B). In a larger prospective study of 88 Quebec hospitals, Hubert *et al* found that among 469 patients, severe CDAD (defined as by *Loo et al*) was higher among patients infected with strains that had both binary toxin and partial *tcdC* deletion [OR = 2.1, 95%CI 0.98 to 4.6, p = 0.054; adjusted for age] [19]. In France, Barbut *et al* conducted a four-year retrospective study and found that among 137 patients, the risk of severe CDAD was higher among patients with binary toxin positive strains [RR = 3.3, 95%CI 1.29 to 8.85, p = 0.01], where severe CDAD was defined as presence of fever, abdominal pain and leukocytosis; or endoscopically or histologically proven pseudomembranous colitis; or toxic megacolon, perforation, colectomy, septic shock or death with *C. difficile* as the primary or contributing factor [20]. In the Netherlands, Goorhuis *et al* compared CDAD patients, of whom 218 had ribotype 027 and 645 had other ribotypes, between February 2005 and November 2006 [23]. Patients with ribotype 027 had more severe diarrhoea (OR = 1.99, 95%CI 0.83 to 4.73), higher attributable mortality (OR = 3.30, 95%CI 0.41 to 26.4) and more recurrences (OR = 1.44, 95%CI 0.94 to 2.20), although the authors considered these findings could be explained by bias in the selection of patients and the low response rate (27%) in their study.

Our study had statistical power of 80% to detect a difference of about 20% or greater at the 5% significance level in disease severity among patients with PCR ribotype 027 compared to other strains. To attribute the increasing incidence of *C. difficile* England and other industrialised countries to a more virulent 027 strain, we would expect it to cause severe disease in at least 20% or more of patients. That we were unable to detect such a difference in severity of CDAD in the 027 versus other ribotypes raises the question of whether this strain can explain recent changes in the epidemiology of *C. difficile* infection. Alternative explanations may include greater risk of transmission of toxigenic strains within health care facilities associated with sub-optimal hygiene [24], greater patient susceptibility associated with prolific use of antibiotics, and an increasingly elderly or vulnerable patient population [25], [26]. It is also likely that some of the reported increase is due to surveillance artefact, reflecting more sensitive and specific tests for *C. difficile* toxins A and B and more complete reporting of cases.

### Conclusions

We did not find evidence to suggest that patients infected with *C. difficile* PCR ribotype 027 were more likely to have severe disease than patients infected with other PCR ribotypes. This finding does not support claims that the emergence of ribotype 027 infections can explain reported increases in incidence of *C. difficile* infections in England. Our results may have relevance to other countries in which virulence associated with the emergence of the 027 ribotype has also been suggested as an explanation for increased incidence of *C. difficile* infections.
